# Association of Sleep Duration With All-Cause and Cardiovascular Mortality: A Prospective Cohort Study

**DOI:** 10.3389/fpubh.2022.880276

**Published:** 2022-07-15

**Authors:** Qiman Jin, Niannian Yang, Juan Dai, Yuanyuan Zhao, Xiaoxia Zhang, Jiawei Yin, Yaqiong Yan

**Affiliations:** ^1^Wuhan Center for Disease Control and Prevention, Wuhan, China; ^2^Department of Nutrition and Food Hygiene, Hubei Key Laboratory of Food Nutrition and Safety, School of Public Health, Tongji Medical College, Huazhong University of Science and Technology, Wuhan, China; ^3^MOE Key Lab of Environment and Health, School of Public Health, Tongji Medical College, Huazhong University of Science and Technology, Wuhan, China

**Keywords:** sleep duration, all–cause mortality, cardiovascular mortality, population attributable fraction, cardiovascular risk

## Abstract

To clarify the association of sleep duration with all-cause and cardiovascular mortality, and further estimate the population attributable fraction (PAF) for the 10-year risk of cardiovascular disease (CVD) due to inappropriate sleep duration among US adults, we included data of the National Health and Nutrition Examination Survey (NHANES) from 2005 to 2014 by linkage to the National Death Index until December 31, 2015 in a prospective design. Cox proportional hazards models were used for multivariate longitudinal analyses. The Pooled Cohort Equations methods was adopted to calculate the predicted 10-year CVD risk. In the current study, sleep <5 h or longer than 9 h per day were significantly associated with elevated risks of all-cause mortality, and the multivariable-adjusted HRs across categories were 1.40 (95% CI, 1.14–1.71), 1.12 (95% CI, 0.91–1.38), 1 (reference), 1.35 (95% CI, 1.12–1.63), and 1.74 (95% CI, 1.42–2.12). Similarly, the HRs of cardiovascular mortality across categories were 1.66 (95% CI, 1.02–2.72), 1.15 (95% CI, 0.77–1.73), 1 (reference), 1.55 (95% CI, 1.05–2.29), and 1.81 (95% CI, 1.09–3.02). Under a causal–effect assumption, we estimated that 187 000 CVD events (PAF 1.8%, 0.9% to 2.3%) were attributable to short sleep duration and 947 000 CVD events (PAF 9.2%, 6.4% to 11.6%) were attributable to long sleep duration from 2018 to 2028. This study informed the potential benefit of optimizing the sleep duration for the primary prevention of CVD in a contemporary population.

## Introduction

Sleep is a complex set of brain processes that supports several physiological needs and healthy sleep duration has been increasingly recognized as an important public health issue (1). The American Academy of Sleep Medicine and the Sleep Research Society recommends that adults should obtain seven or more hours of sleep per night to promote optimal health. However, the proportion of US adults with short sleep duration (< 7 h/ day) have gradually increased over the past decades; while the prevalence of long sleep duration (defined as ≥ 9 h) shows an opposite trend (2, 3). Although experimental findings strongly indicate that short sleep duration is associated with adverse physiological and immunological consequences, several researchers suggesting that long sleep may be a greater concern than short sleep duration (4).

A previous meta–analysis suggested that both short and long sleep durations were associated with an increased risk of all–cause mortality and cardiovascular events (5), however, others did not find consistent results (6, 7). The interpretation of the reported associations between sleep duration and mortality has been restricted by various study design (cross–sectional, case–control or prospective design), diverse target populations (general population or subjects with metabolic disease), and differences in the reference sleep duration (6–8 h, 7–8 h, or 7–9 h). Therefore, there is a need for well–designed studies to confirm the relationship. In addition, cardiovascular disease (CVD) is the leading cause of death in the United States (8). However, no study has translated a prospective association between sleep duration and cardiovascular health into a measure of its contemporary population level impact, including population attributable fraction.

In this study, we used data collected from the National Health and Nutrition Examination Surveys (NHANES) linked to National Death Index records to clarify the association of sleep duration with all–cause and cardiovascular mortality. Moreover, to provide policy relevant measures, we further estimated the population attributable fraction for the 10–year risk of developing cardiovascular events due to short/long sleep duration in contemporary populations of the US.

## Methods

### Study Design and Population

NHANES is an ongoing national cross–sectional survey administered by the National Center for Health Statistics (NCHS) to assess the health and nutritional status of the civilian population in the United States since 1999 (9). Research Ethics Review Board approved the underlying protocol and written informed consent was obtained from all participants. Details on NHANES study design, study protocol, and data collection have been described elsewhere (10). The questions on sleep duration were added to NHANES from 2005. We included data on adults (≥ 20 years) during the 5 cycles of the National Health and Nutrition Examination Survey (NHANES) from 2005 to 2014 by linkage to the National Death Index (until December 31, 2015) in a prospective design (Supplementary Figure S1). In addition, we further estimated the population attributable fraction for the 10–year risk of developing cardiovascular events due to short/long sleep duration from the most recent cycle of NHANES (2017–2018), which could provide the newest estimates (Supplementary Figure S1).

### Sleep Duration Assessment

During the 5 cycles of NHANES from 2005 to 2014, sleep duration was determined using the question, “How much sleep do you usually get at night on weekdays or workdays?” The response was recorded as the number of hours of sleep obtained on a typical night, which was assessed as a continuous variable. In the cycle of NHANES (2017–2018), two questions were asked, “What time do you usually fall asleep on weekdays or workdays?”, and “What time do you usually wake up on weekdays or workdays”. Hours of sleep on weekdays or workdays were calculated from the reported usual sleep time and wake time during main sleeping period.

### Ascertainment of Mortality

All–cause and CVD mortality were determined by linking to the National Death Index until December 31, 2015. The anonymized data of serial NHANES 2005–2014 were linked to mortality data with the assigned sequence number. Death from CVD was defined as codes I00–I09, I11, I13, I20–I51, and I60–I69 using the International Statistical Classification of Diseases and Related Health Problems, Tenth Revision (ICD−10). The follow–up time was identified as the period between the date of interview and the date of mortality or the end of follow–up (December 31, 2015), whichever occurred first.

### Covariates

The study covariates included demographic characteristics and lifestyle factors. Age, sex, race/ethnicity, educational level, smoking status, physical activity, alcohol consumption, body weight, and height were collected during interviews. Smokers were defined as individuals who reported smoking at least 100 cigarettes during their lifetime. Drinkers were defined as participants who drank at least 12 drinks of alcohol in any given year. Participants with more than 2.5 h of exercise per week were classified as physically active. Diet quality was assessed by Healthy Eating Index 2015 (HEI−2015) and the methods have been described previously (11). Comorbidity conditions were determined to be diagnosed and/or to take prescribed medications for the disease. Family history of chronic disease (heart disease and diabetes) were defined if participants reported that their close relatives had such diseases.

### Statistical Analysis

Sleep duration was categorized into five categories (≤ 5, 6, 7, 8, and ≥ 9 h). Cox proportional hazards models were used to estimate hazard ratios (HRs) and 95% CIs with 7 h regarded as reference. Model 1 was adjusted for age and sex. Model 2 was further adjusted for race/ethnicity, body mass index (BMI), educational level, physical activity, alcohol consumption, smoking status, and diet quality based on model 1. Model 3 was further adjusted for family history of chronic disease and comorbidity conditions based on model 2. To describe the potential non–linear association between sleep duration and all–cause mortality, cox proportional hazard models were used with sleep duration treated as continuous variables and fitted on restricted cubic splines with 3 knots. Likelihood ratio tests were utilized to examine the non–linearity of the exposures. We further applied stratification analysis for associations of sleep duration with all–cause and cardiovascular mortality according to several potential confounding factors such as age (younger or older than 65 years), sex, race/ethnicity (non–Hispanic white or other), education (less than college or college or above), BMI (< 30 or ≥ 30), smoking (never smoker or ever smoker), alcohol drinking (never drinker or ever drinker), physical activity (physically inactive or physically active). The survey–weighted Wald F statistic was used to evaluate the interaction effect between sleep duration and subgroup variables, and the *P*–value for multiple testing was adjusted using the Bonferroni correction with statistical significance set at *P* < 0.005 (0.05/8 [subgroups]). Several sensitivity analyses were performed by (1) excluding the participants with heart disease or cancer; (2) excluding the participants died in the first year of follow–up.

Under an assumption of causality, we estimated the proportion of CVD events attributable to short/long sleep duration in the US. Sleep duration and risk factors for CVD were basing on the data from the NHANES 2017–2018 cycle (12). Pregnant women and subjects with self–reported CVD were excluded from the analysis (13). The American Heart Association released a report that presented updated risk equations, the Pooled Cohort Equations (PCE), for CVD (13). The PCE provide estimates of the 10–year risk of CVD for men and women 40 to 79 years of age. The variables that statistically merit inclusion in the risk assessment equations are age, total cholesterol, high–density lipoprotein cholesterol, systolic blood pressure (including treated or untreated status), diabetes mellitus, and current smoking status. The 10–year risk estimate for CVD was calculated through a series of steps. The natural log of age, total cholesterol, high–density lipoprotein cholesterol, and systolic blood pressure were first calculated with systolic blood pressure being either a treated or untreated value. Any appropriate interaction terms were then calculated. These values were then multiplied by the coefficients from the equation for the specific race–sex group of the individual. The sum of the “Coefficient × Individual Value” was then calculated for the individual. The estimated 10–year risk of a first CVD event (R_p_) is formally calculated as 1 minus the survival rate at 10 years, raised to the power of the exponent of the “Coefficient × Value” sum minus the race– and sex–specific overall mean “Coefficient × Value” sum. The risk equation has been validated and showed to be well calibrated in the US population (14, 15). The altered predicted 10–year risk for each person (R_q_) by adjusting the sleep duration to 7 h was evaluated. Furthermore, the risk attributable to short/long sleep duration was calculated with the value of (R_p_−*R*_q_). If one adult slept *x* hours longer/shorter than 7 hours, the risk for the individual (*RR*_*x*_) could be calculated as follows: *RR*_*x*_ = e^lnHR^^*^x, where the HR of per hour increment (>7 h per day, HR 1.12 95% CI 1.06 to 1.16) and per hour decrement (<7 h per day, HR 1.06 95% CI 1.03 to 1.08) from a most comprehensive meta–analysis (5). If the participant's sleep duration became 7 h, the altered predicted 10–year risk (R_q_) would be R_p_/*RR*_*x*_ (16). We estimated Σ(R_p_−*R*_q_) × population size as a number of CVD events attributable to sleep duration in the US. In addition, population attributable fraction was derived as Σ (R_p_−*R*_q_)/ Σ (R_p_) (17). Detail description of the analysis has been described in our previous publication (18).

All analyses incorporated sample weights, stratification, and clustering of the complex sampling design to ensure nationally representative estimates. SAS version 9.4 (SAS Institute Inc) and Stata version 14 (StataCorp LP, TX, USA) were used for the analysis. Statistical significance was set at a 2–tailed *P* < 0.05.

## Results

### Participants Characteristics

Of the 25 481 US adults, 12 388 (48.6%) were men (mean age, 46.3 years) and 13 093 (51.4%) were women (mean age, 47.8 years). During 146 484 person–years of follow–up, we documented 2033 deaths due to all causes, including 378 deaths due to heart disease and 461 deaths due to cancer. Table 1 showed the baseline characteristics of participants according to sleep duration. Compared with the subjects with sleep duration of 7 h/day, slept more than 9 h/day were more often older, female, non-smoker, non-drinker, physically inactive, were less educated (Table 1). Participants with an estimated sleep duration of ≤ 5 h were more likely to be minorities, non-smoker, non-drinker, physically inactive, less educated, and had higher BMI, and poor diet quality. There were more participants with extreme sleep duration reporting family history of chronic disease and having morbidity conditions, such as diabetes, heart disease, and cancer.

**Table 1:** Characteristics of study participants by categories of sleep duration^*^.

	**Sleep duration (hours)**				
	**≤5**	**6**	**7**	**8**	**≥9**
Participants	3947 (13.17)	5999 (23.04)	6777 (29.11)	6761 (27.02)	1997 (7.66)
Age, mean (SD), years	46.40 (15.83)	45.97 (16.03)	46.95 (16.34)	47.93 (17.70)	49.26 (20.18)
BMI, mean (SD), kg/m^2^	30.05 (7.68)	29.09 (6.85)	28.44 (6.32)	28.35 (6.58)	28.40 (7.02)
Male	1945 (48.34)	2996 (51.15)	3383 (50.19)	3181 (45.70)	883 (39.41)
Race/ethnicity					
Non-Hispanic white	1440 (58.60)	2495 (65.21)	3426 (73.36)	3253 (70.88)	1041 (70.93)
Non-Hispanic black	1326 (21.16)	1484 (13.66)	1051 (7.39)	1200 (9.25)	382 (10.72)
Hispanic	893 (13.21)	1488 (14.04)	1642 (12.38)	1791 (13.98)	449 (12.86)
Other	288 (7.03)	532 (7.09)	658 (6.87)	517 (5.88)	125 (5.49)
Educational level					
Less than high school	1181 (22.53)	1424 (15.74)	1517 (14.08)	1875 (17.93)	646 (23.52)
High school graduate or GED	1013 (27.75)	1451 (24.99)	1429 (20.31)	1546 (22.68)	473 (22.81)
Some college or above	1753 (49.72)	3124 (59.28)	3831 (65.61)	3340 (59.39)	878 (53.67)
Smoker	1895 (45.32)	3266 (53.50)	3867 (57.69)	3787 (54.99)	1045 (52.07)
Drinker	2536 (68.80)	4126 (74.07)	4709 (74.57)	4493 (72.56)	1277 (68.44)
HEI-2015 scores	55.52 (8.53)	56.84 (8.73)	58.00 (8.85)	58.22 (8.77)	57.47 (8.46)
Physically active	949 (26.33)	1774 (34.13)	2173 (37.04)	1962 (34.74)	476 (28.82)
History of diabetes	614 (11.66)	745 (9.20)	655 (7.18)	817 (8.94)	333 (11.73)
History of heart disease	564 (11.39)	568 (7.41)	553 (6.29)	714 (8.93)	340 (12.86)
History of cancer	346 (9.16)	499 (8.10)	591 (9.34)	682 (10.73)	257 (12.20)
Family history of diabetes	1856 (44.07)	2507 (38.79)	2574 (35.25)	2522 (35.15)	747 (34.18)
Family history of heart disease	647 (17.57)	749 (13.50)	747 (11.20)	738 (12.33)	227 (13.00)

*BMI, body mass index (calculated as weight in kilograms divided by height in meters squared); GED, General Educational Development; HEI, Healthy Eating Index; SD, standard deviation*.

* *Data are presented as number (percentage) of study participants unless otherwise indicated*.

### Sleep Duration and All–Cause Mortality

**Figure 1:**
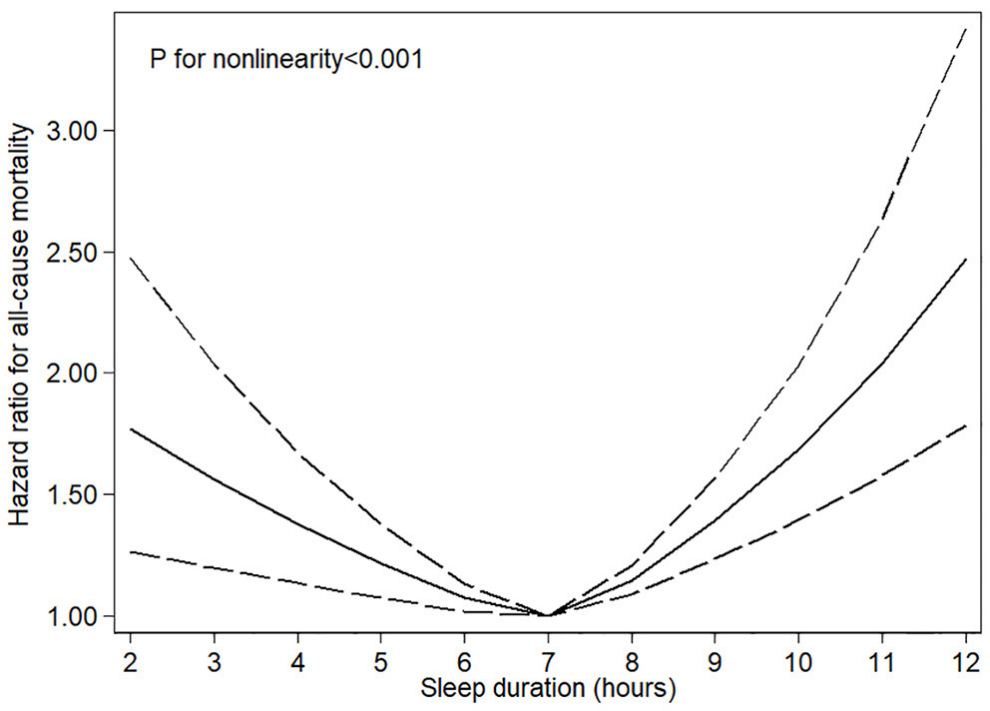
Restricted cubic spline models for sleep duration and risk of all–cause mortality. Knots were placed at the 10th, 50th, and 90th percentiles of sleep duration. Results were adjusted for age, sex, race/ethnicity, education, physical activity, drinking and smoking status, HEI−2015 scores, body mass index, family history of diabetes mellitus and heart disease, history of diabetes, heart disease, and cancer.

As shown in Table 2, after adjustment for age and sex, sleep < 5 h and more than 9 h per day were associated with significant elevated risks of all–cause mortality, and the HRs across categories were 1.79 (95% CI, 1.47–2.18), 1.20 (95% CI, 0.99–1.45), 1 (reference), 1.44 (95% CI, 1.18–1.75), and 2.06 (95% CI, 1.67–2.53). In the multivariable models, the association was slightly attenuated but a significant U–shaped relationship of sleep duration with all–cause mortality was still observed, and the multivariable–adjusted HRs across categories were 1.40 (95% CI, 1.14–1.71), 1.12 (95% CI, 0.91–1.38), 1 (reference), 1.35 (95% CI, 1.12–1.63), and 1.74 (95% CI, 1.42–2.12). In the restrict cubic spline, significant nonlinear association was observed between sleep duration and all–cause mortality (*P* < 0.001) (Figure 1).

**Table 2:** Hazard ratios (95% CIs) of all-cause and cardiovascular mortality according to sleep duration in NHANES.

	**Hazard ratio (95% CI)**				
	**≤ 5**	**6**	**7**	**8**	**≥9**
Person-years	22731	34409	39595	38858	10891
All-cause mortality				
No. of cases	340	386	406	615	286
Model 1	1.79 (1.47 to 2.18)	1.20 (0.99 to 1.45)	1	1.44 (1.18 to 1.75)	2.06 (1.67 to 2.53)
Model 2	1.46 (1.18 to 1.79)	1.46 (1.18 to 1.79)	1.46	1.46 (1.18 to 1.79)	1.46 (1.18 to 1.79)
Model 3	1.40 (1.14 to 1.71)	1.12 (0.91 to 1.38)	1.00	1.35 (1.12 to 1.63)	1.74 (1.42 to 2.12)
Cardiovascular mortality				
No. of cases	60	66	72	121	59
Model 1	2.09 (1.28 to 3.39)	1.22 (0.83 to 1.81)	1	1.62 (1.10 to 2.39)	2.11 (1.28 to 3.45)
Model 2	1.75 (1.06 to 2.89)	1.16 (0.78 to 1.73)	1.00	1.56 (1.06 to 2.28)	1.86 (1.11 to 3.13)
Model 3	1.66 (1.02 to 2.72)	1.15 (0.77 to 1.73)	1.00	1.55 (1.05 to 2.29)	1.81 (1.09 to 3.02)

*Model 1: adjusted for age (continuous), sex (male or female)*.

*Model 2: further adjusted for race/ethnicity (non-Hispanic white, non-Hispanic black, Hispanic, and other), body mass index (<21, 21–24.9, 25–29.9, 30–35, and ≥35), educational level (less than high school, high school graduate or General Educational Development, and some college or above), physical activity (0, 0.1–0.9, 1.0–3.4, 3.5–5.9, or ≥6 h per week), drinking status (yes, no), smoking status (never smoker, former smoker, or current smoker), and HEI-2015 (continuous)*.

*Model 3: further adjusted for family history of diabetes mellitus (yes or no), family history of heart disease (yes or no), history of diabetes (yes or no), history of heart disease (yes or no), history of cancer (yes or no)*.

### Sleep Duration and Cardiovascular Mortality

**Figure 2:**
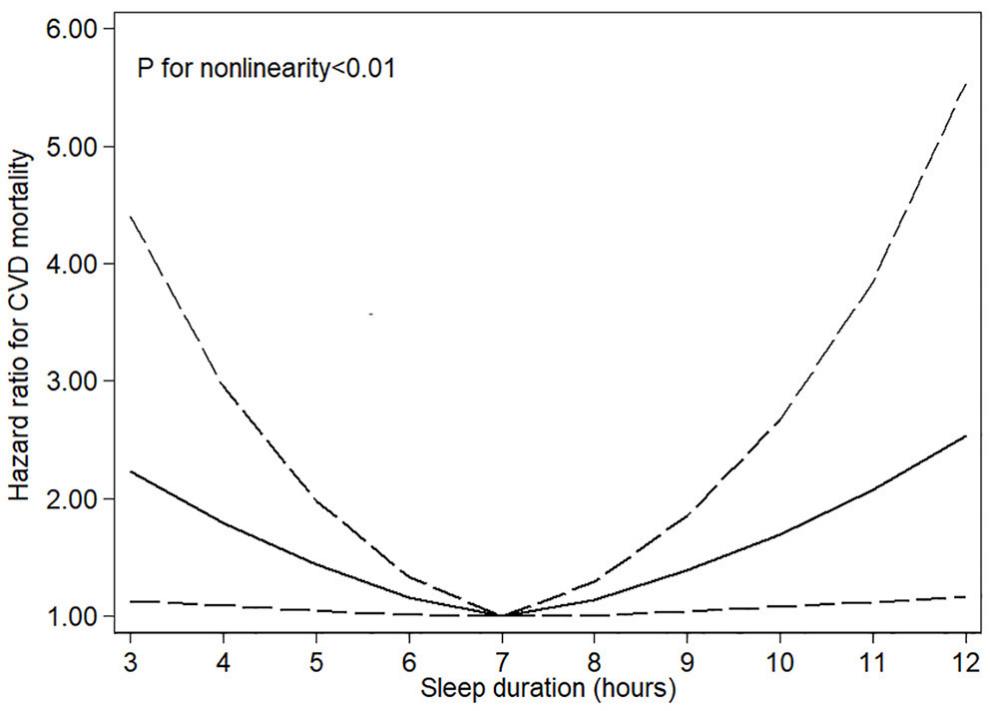
Restricted cubic spline models for sleep duration and risk of cardiovascular mortality. Knots were placed at the 10th, 50th, and 90th percentiles of sleep duration. Results were adjusted for age, sex, race/ethnicity, education, physical activity, drinking and smoking status, HEI−2015 scores, body mass index, family history of diabetes mellitus and heart disease, history of diabetes, heart disease, and cancer.

As shown in Table 2, after adjustment for age and sex, sleep < 5 h and more than 9 h per day were associated with significant elevated risk of cardiovascular mortality, and the HRs across categories were 2.09 (95% CI, 1.28–3.39), 1.22 (95% CI, 0.83–1.81), 1 (reference), 1.62 (95% CI, 1.10–2.39), and 2.11 (95% CI, 1.28–3.45). In the fully adjusted models, the associations were largely attenuated, and the multivariable–adjusted HRs across categories were 1.66 (95% CI, 1.02–2.72), 1.15 (95% CI, 0.77–1.73), 1 (reference), 1.55 (95% CI, 1.05–2.29), and 1.81 (95% CI, 1.09–3.02). Significant non-linear association was observed between sleep duration and cardiovascular mortality (*P* < 0.01) (Figure 2).

### Subgroup and Sensitivity Analyses

In subgroup analysis, the results remained consistent in most subgroups and there was no significant interaction observed in each subgroup (Supplementary Tables S1, S2 in the Supplementary). In sensitivity analyses, the results remained similar after excluding deaths during the first year (Supplementary Table S3 in the Supplementary), and excluding subjects with history of heart disease or cancer at baseline (Supplementary Table S4 in the Supplementary).

### Cardiovascular Disease Risk Attributable to Sleep Duration

We analyzed 1805 subjects aged 40 to 79 years in the National Health and Nutrition Examination Survey, 2017–2018 cycle. After accounting for the nationally representative, complex, stratified, multistage design and the sample weight, approximately 112.8 million adults were represented. The absolute event rates were estimated to be 9.2% (10.3 million CVD events) in the US from 2018 to 2028. Under an assumption of causality, 187 000 CVD events over 10 years (population attributable fraction 1.8%, 0.9% to 2.3%) were estimated to be attributable to short sleep duration and 947 000 CVD events (population attributable fraction 9.2%, 6.4% to 11.6%) were estimated to be attributable to long sleep duration (Table 3). Adults younger than 60 years, men, and the non–Hispanic black would have a greater proportion of CVD events related to short sleep duration, while older adults (60–79 years), women, and other races would have a greater proportion of CVD events related to long sleep duration.

**Table 3:** The estimated population attributable fraction for cardiovascular disease risk from short and long sleep duration in the United States.

**Population**	Adults free of CVD, N/1,000 (40–79 years)^*^	**Sleep duration, Mean ±SE, hours/day**	**CVD in 10 years, n/1,000 (10-year risk)^‡^**	**CVD events attributable to sleep duration**			
				**Short sleep duration**		**Long sleep duration**	
				**n/1,000**	**PAF (95% CI)**	**n/1,000**	**PAF (95% CI)**
All	112,780	7.44 ± 0.06	10,327 (9.2)	187	1.8 (0.9 to 2.3)	947	9.2 (6.4 to 11.6)
Sex							
Male	52,658	7.25 ± 0.06	5,991 (11.4)	124	2.1 (1.1 to 1.7)	484	8.1 (5.7 to 10.2)
Female	60,122	7.61 ± 0.07	4,337 (7.2)	63	1.5 (0.8 to 1.9)	462	10.7 (7.5 to 13.5)
Age							
40–59	70,027	7.34 ± 0.06	3,061 (4.4)	75	2.5 (1.3 to 3.2)	210	6.9 (4.8 to 8.7)
60–79	42,753	7.62 ±0.10	7,267 (17.0)	112	1.5 (0.8 to 2.0)	737	10.1 (7.1 to 12.8)
Race							
Hispanic	15,830	7.43 ± 0.07	1,240 (7.8)	23	1.9 (1.0 to 2.4)	126	10.2 (7.2 to 12.8)
Non-Hispanic white	74,515	7.48 ± 0.08	6,812 (9.1)	104	1.5 (0.8 to 2.0)	628	9.2 (6.5 to 11.7)
Non-Hispanic black	10,960	7.25 ± 0.06	1,294 (11.8)	38	2.9 (1.5 to 3.8)	109	8.4 (6.0 to 10.7)
Others	11,475	7.43 ± 0.07	981 (8.6)	23	2.3 (1.2 to 3.1)	83	8.5 (5.9 to 10.7)

*CVD, cardiovascular disease; PAF, population attributable fraction; SE, standard error*.

* *The number of subjects was estimated in National Health and Nutrition Examination Survey in the United States, 2017–2018 cycle. N representing 112.8 million men and non-pregnant women who were aged 40 to 79 years and free of self-reported cardiovascular disease (congestive heart failure, coronary heart disease, angina, myocardial infarction, or stroke)*.

‡ *10-year risk of cardiovascular disease was predicted using the Pooled Cohort Equations in each of the United States*.

## Discussion

In this population–based cohort study, U–shaped associations of sleep duration with all–cause and cardiovascular mortality were observed with the lowest risk at 7 h/day of sleep. Under assumption of causality for the association of sleep duration with incidence of CVD, we provided efficacy estimates that 187 000 and 957 000 CVD events over 10 years would be related to short sleep duration and long sleep duration, respectively.

The association of short sleep duration with all–cause mortality and CVD mortality was relatively weaker in our analysis, which was in accordance with the Prospective Urban Rural Epidemiology (PURE) study (19), and the Shanghai Women's and Men's Health Studies (20). Considering an increasing number of Americans choose to curtail sleep in favor of other social, leisure, or work–related activities, the recommended sleep duration is at least 7 h per day for metabolic health (21). However, what an upper limit of sleep duration for optimal health remains unclear. In our results, we observed that sleep more than 9 h per day had a significantly positive association with all–cause and CVD–cause mortality, which was in accordance with previous finding (5). It is possible that those with longer sleep duration may be underlying subclinical health status, and extreme long sleep might be a marker of chronic disease. Health status may be crucial confounding for the association between sleep duration and mortality. However, in our sensitivity analyses, excluding the participants with a history of heart disease or cancer at baseline resulted in consistent findings. Furthermore, after excluding the subjects died within the first year of follow up also did not alter the results. The robust results suggest that sleep duration *per se* may be related with elevated mortality risks.

Although causality has not been established, our findings and available evidence indicate a benefit of optimizing the sleep duration for the primary prevention of CVD. While previous study had elucidated that mean–predicted 10–year CVD risk was lowest among adults who reported sleeping 7 h per night (22), no study examined the population impact by combining estimates for sleep duration among national represented populations and provided quantitative evidence on the association of sleep duration with incidence of CVD. The current habitual sleep duration, especially long sleep duration, was estimated to cause approximately one million excess events of CVD in the US over 10 years. Although it is crucial to emphasize the regular basis of sleep (seven or more hours) for adults in modern societies, the potential harmful effect of long sleep duration still need to be paid attention to.

Several potential mechanisms may explain the relationship between sleep duration and mortality. Previous studies have showed that, sleep restriction could influence the endocrine and metabolic system, such as decreased the levels of testosterone and melatonin (23, 24), reduced level of leptin, and elevated level of ghrelin (25, 26), which may be implicated with mortality (27–30). The mechanisms for extreme long sleep duration and mortality are considered more indeterminate. Besides the confounding effects of sub–healthy status or uncontrolled chronic illness, sleep fragmentation, linked with excessive sleep (31), was found to be associated with arteriolosclerosis (32). Moreover, long periods of sleep were linked with fatigue and lethargy, which in turn could lead to prolonged sleep. These states may not provide adequate recovery from stress and illness, which then led to increased mortality (33). Further experimental studies are needed to explore the potential impact of sleep extension on health outcomes in future.

Our study has several limitations. First, the sleep duration was based on self–report interview and the subject misclassifications might occur. However, objective measurement of sleep duration is not feasible in large epidemiological studies, and the subjective estimates of sleep duration are highly correlated with objective measurements like polysomnography (34). Second, a single measure of sleep duration at baseline may not fully represent the sleep habits over time when relating them to long–term disease incidence. Third, we cannot completely rule out the residual or unmeasured confounding despite we had adjusted extensively for the major confounding factors. Forth, under the assumption of causality, we estimated the population attributable fraction of sleep duration based on an observational study design. Ideally large randomized trials of different sleep durations were still limited, and the feasible study design was a challenge. Thus, results from large cohort studies are still the best evidence to evaluate the longitudinal effect of sleep duration on mortality at this stage.

## Conclusion

Our study showed U–shaped associations of sleep duration with all–cause and cardiovascular mortality, and 7 h sleep duration was associated with the lowest risk. This study also informs the potential benefit of optimizing the sleep duration for the primary prevention of CVD in a contemporary population.

## Data Availability Statement

The original contributions presented in the study are included in the article/Supplementary Material, further inquiries can be directed to the corresponding authors.

## Ethics Statement

The studies involving human participants were reviewed and approved by Research Ethics Review Board of the National Health and Nutrition Examination Survey. The patients/participants provided their written informed consent to participate in this study.

## Author Contributions

YY and JY conceived and designed the study, supervised the study, provided administrative, technical, and material support. QJ drafted the article. NY, JD, YZ, XZ, and JY critically revised the article. JY and QJ had full access to all the data in the study, takes responsibility for the integrity of the data, the accuracy of the data analysis, and the statistical analysis. QJ, NY, JD, YZ, XZ, JY, and YY acquired, analyzed, interpreted the data, and critically revised the article for important intellectual content. All authors contributed to the article and approved the submitted version.

## Funding

This work was funded by grant 82103832 from the Young Scientists Fund of the National Natural Science Foundation of China. The funders had no role in study design, data collection and analysis, decision to publish, and preparation of the manuscript.

## Conflict of Interest

The authors declare that the research was conducted in the absence of any commercial or financial relationships that could be construed as a potential conflict of interest.

## Publisher's Note

All claims expressed in this article are solely those of the authors and do not necessarily represent those of their affiliated organizations, or those of the publisher, the editors and the reviewers. Any product that may be evaluated in this article, or claim that may be made by its manufacturer, is not guaranteed or endorsed by the publisher.

## Abbreviations

BMI: body mass index
CVD: cardiovascular disease
HEI-2015: Healthy Eating Index 2015
HR: hazard ratios
ICD: International Statistical Classification of Diseases and Related Health Problems
NCHS: National Center for Health Statistics
NHANES: National Health and Nutrition Examination Surveys
PURE: Prospective Urban Rural Epidemiology study.
