# Supplementary material for: Association of Sleep Duration With All-Cause and Cardiovascular Mortality: A Prospective Cohort Study
Source: Front Public Health. 2022 Jul 15;10:880276. doi: 10.3389/fpubh.2022.880276 (PMC9334887; doi:10.3389/fpubh.2022.880276)
Supplement: Supplementary file 1 [file Data_Sheet_1.docx]

Supplementary material

Association of Sleep Duration with All-cause and Cardiovascular Mortality: A Prospective Cohort Study

Qiman Jin, Niannian Yang, Juan Dai, Yuanyuan Zhao, Xiaoxia Zhang, Jiawei Yin, Yaqiong Yan

Table S1 Hazard ratios (95% CIs) of all-cause mortality according to sleep duration by subgroups in NHANES.

Table S2 Hazard ratios (95% CIs) of cardiovascular mortality according to sleep duration by subgroups in NHANES.

Table S3 Sensitivity analysis of all-cause and cause-specific mortality according to sleep duration in NHANES by excluding the participants with a history of heart disease or cancer.

Table S4 Sensitivity analysis of all-cause and cause-specific mortality according to sleep duration in NHANES by excluding the participants who died during the first year of follow-up.

Figure S1 Flow chart of the study design.

Table S1 Hazard ratios (95% CIs) of all-cause mortality according to sleep duration by subgroups in NHANES.

|  | **Hazard ratio (95% CI)** | | | | | ***P* for interaction** |
| --- | --- | --- | --- | --- | --- | --- |
|  | **≤5** | **6** | **7** | **8** | **≥9** |  |
| Age, y |  |  |  |  |  |  |
| <65 | 1.22 (0.81 to 1.84) | 1.09 (0.77 to 1.54) | 1.00 | 1.23 (0.87 to 1.73) | 1.68 (1.05 to 2.68) | 0.62 |
| ≥65 | 1.44 (1.16 to 1.79) | 1.15 (0.95 to 1.39) | 1.00 | 1.34 (1.11 to 1.61) | 1.61 (1.30 to 1.99) |  |
| Sex |  |  |  |  |  |  |
| Male | 1.63 (1.24 to 2.13) | 1.19 (0.94 to 1.51) | 1.00 | 1.57 (1.31 to 1.89) | 1.80 (1.41 to 2.29) | 0.36 |
| Female | 1.16 (0.84 to 1.59) | 1.06 (0.79 to 1.42) | 1.00 | 1.18 (0.89 to 1.57) | 1.73 (1.28 to 2.35) |  |
| Race/ethnicity |  |  |  |  |  |  |
| Non-Hispanic white | 1.65 (1.27 to 2.14) | 1.22 (0.93 to 1.59) | 1.00 | 1.47 (1.18 to 1.85) | 1.97 (1.57 to 2.49) | 0.46 |
| Other | 0.95 (0.74 to 1.20) | 0.91 (0.70 to 1.19) | 1.00 | 1.03 (0.82 to 1.28) | 1.11 (0.84 to 1.48) |  |
| Educational level |  |  |  |  |  |  |
| Less than college | 1.32 (1.05 to 1.67) | 1.14 (0.90 to 1.46) | 1.00 | 1.37 (1.10 to 1.69) | 1.78 (1.37 to 2.31) | 0.57 |
| College or above | 1.53 (1.08 to 2.16) | 1.05 (0.80 to 1.39) | 1.00 | 1.33 (1.01 to 1.75) | 1.71 (1.22 to 2.39) |  |
| BMI |  |  |  |  |  |  |
| <30 | 1.37 (1.06 to 1.77) | 1.14 (0.90 to 1.46) | 1.00 | 1.37 (1.08 to 1.73) | 1.72 (1.29 to 2.31) | 0.32 |
| ≥30 | 1.48 (1.01 to 2.18) | 1.11 (0.81 to 1.53) | 1.00 | 1.31 (0.97 to 1.77) | 1.76 (1.23 to 2.52) |  |
| Smoking status |  |  |  |  |  |  |
| Never smoker | 1.51 (1.07 to 2.13) | 1.38 (0.99 to 1.92) | 1.00 | 1.44 (1.04 to 1.99) | 2.08 (1.47 to 2.93) | 0.33 |
| Ever smoker | 1.31 (1.00 to 1.70) | 0.95 (0.74 to 1.22) | 1.00 | 1.27 (1.01 to 1.59) | 1.46 (1.15 to 1.86) |  |
| Alcohol use |  |  |  |  |  |  |
| Never drinker | 1.35 (0.99 to 1.85) | 0.96 (0.73 to 1.25) | 1.00 | 1.29 (1.00 to 1.67) | 1.44 (1.06 to 1.95) | 0.20 |
| Ever drinker | 1.48 (1.07 to 2.04) | 1.48 (1.08 to 2.04) | 1.00 | 1.51 (1.14 to 2.00) | 2.31 (1.58 to 3.36) |  |
| Physical activity |  |  |  |  |  |  |
| Physically inactive | 1.34 (0.74 to 2.43) | 0.99 (0.59 to 1.65) | 1.00 | 1.40 (0.95 to 2.07) | 0.90 (0.48 to 1.70) | 0.21 |
| Physically active | 1.48 (1.22 to 1.79) | 1.17 (0.95 to 1.44) | 1.00 | 1.37 (1.13 to 1.67) | 1.95 (1.59 to 2.38) |  |

^a^ Adjusted for age (continuous), sex (male or female), race/ethnicity (non-Hispanic white, non-Hispanic black, Hispanic, and other), educational level (less than high school, high school graduate or General Educational Development, and some college or above), family history of diabetes mellitus (yes or no), family history of heart disease (yes or no), history of diabetes (yes or no), history of heart disease (yes or no), history of cancer (yes or no), physical activity (0, 0.1-0.9, 1.0-3.4, 3.5-5.9, or ≥6 hours per week), drinking status (yes, no), smoking status (never smoker, former smoker, or current smoker), HEI-2015 scores (continuous), and body mass index (<21, 21-24.9, 25-29.9, 30-35, and ≥35) except the corresponding subgroup variates.

Table S2 Hazard ratios (95% CIs) of cardiovascular mortality according to sleep duration by subgroups in NHANES.

|  | **Hazard ratio (95% CI)** | | | | | ***P* for interaction** |
| --- | --- | --- | --- | --- | --- | --- |
|  | **≤5** | **6** | **7** | **8** | **≥9** |  |
| Age, y |  |  |  |  |  |  |
| <65 | 1.00 (0.37 to 2.67) | 0.95 (0.46 to 1.96) | 1.00 | 1.09 (0.40 to 3.00) | 1.00 (0.39 to 2.56) | 0.60 |
| ≥65 | 1.95 (1.12 to 3.39) | 1.23 (0.72 to 2.11) | 1.00 | 1.66 (1.05 to 2.62) | 1.93 (1.11 to 3.35) |  |
| Sex |  |  |  |  |  |  |
| Male | 1.77 (0.96 to 3.27) | 1.23 (0.74 to 2.05) | 1.00 | 1.67 (1.14 to 2.45) | 1.72 (0.97 to 3.05) | 0.60 |
| Female | 1.44 (0.68 to 3.05) | 1.04 (0.51 to 2.12) | 1.00 | 1.35 (0.67 to 2.73) | 1.97 (0.91 to 4.25) |  |
| Race/ethnicity |  |  |  |  |  |  |
| Non-Hispanic white | 1.81 (0.99 to 3.30) | 1.15 (0.67 to 1.95) | 1.00 | 1.58 (1.00 to 2.51) | 1.85 (1.02 to 3.34) | 0.85 |
| Other | 1.49 (0.75 to 2.96) | 1.30 (0.65 to 2.62) | 1.00 | 1.42 (0.80 to 2.52) | 1.73 (0.89 to 3.37) |  |
| Educational level |  |  |  |  |  |  |
| Less than college | 1.70 (0.90 to 3.21) | 1.17 (0.71 to 1.93) | 1.00 | 1.59 (1.00 to 2.53) | 1.81 (1.09 to 3.03) | 0.97 |
| College or above | 1.50 (0.71 to 3.18) | 1.04 (0.54 to 1.99) | 1.00 | 1.43 (0.77 to 2.66) | 1.63 (0.74 to 3.60) |  |
| BMI |  |  |  |  |  |  |
| <30 | 1.80 (1.01 to 3.21) | 0.87 (0.52 to 1.46) | 1.00 | 1.26 (0.83 to 1.92) | 1.41 (0.74 to 2.67) | 0.15 |
| ≥30 | 1.45 (0.55 to 3.84) | 2.30 (1.12 to 4.73) | 1.00 | 2.62 (1.28 to 5.38) | 3.73 (1.52 to 9.15) |  |
| Smoking status |  |  |  |  |  |  |
| Never smoker | 3.19 (1.43 to 7.13) | 1.94 (0.82 to 4.59) | 1.00 | 2.65 (1.20 to 5.86) | 2.78 (1.33 to 5.81) | 0.48 |
| Ever smoker | 1.11 (0.56 to 2.20) | 0.86 (0.52 to 1.43) | 1.00 | 1.16 (0.70 to 1.91) | 1.46 (0.77 to 2.80) |  |
| Alcohol use |  |  |  |  |  |  |
| Never drinker | 1.54 (0.79 to 2.98) | 1.06 (0.62 to 1.82) | 1.00 | 1.59 (0.98 to 2.59) | 1.54 (0.70 to 3.39) | 0.92 |
| Ever drinker | 1.90 (0.93 to 3.88) | 1.32 (0.72 to 2.45) | 1.00 | 1.44 (0.79 to 2.63) | 2.38 (1.22 to 4.64) |  |
| Physical activity |  |  |  |  |  |  |
| Physically inactive | 2.13 (0.98 to 4.59) | 1.29 (0.39 to 4.26) | 1.00 | 1.74 (0.72 to 4.23) | 1.65 (0.58 to 4.70) | 0.62 |
| Physically active | 1.67 (0.94 to 2.96) | 1.15 (0.71 to 1.86) | 1.00 | 1.54 (1.01 to 2.35) | 1.91 (1.11 to 3.30) |  |

^a^ Adjusted for age (continuous), sex (male or female), race/ethnicity (non-Hispanic white, non-Hispanic black, Hispanic, and other), educational level (less than high school, high school graduate or General Educational Development, and some college or above), family history of diabetes mellitus (yes or no), family history of heart disease (yes or no), history of diabetes (yes or no), history of heart disease (yes or no), history of cancer (yes or no), physical activity (0, 0.1-0.9, 1.0-3.4, 3.5-5.9, or ≥6 hours per week), drinking status (yes, no), smoking status (never smoker, former smoker, or current smoker), HEI-2015 scores (continuous), and body mass index (<21, 21-24.9, 25-29.9, 30-35, and ≥35) except the corresponding subgroup variates.

Table S3 Sensitivity analysis of all-cause and cause-specific mortality according to sleep duration in NHANES by excluding the participants with a history of heart disease or cancer.

|  | **Hazard ratio (95% CI)** | | | | |
| --- | --- | --- | --- | --- | --- |
|  | ≤5 | 6 | 7 | 8 | ≥9 |
| Person-years | 22713 | 34385 | 39571 | 38825 | 10865 |
| All-cause mortality |  |  |  |  |  |
| No. of cases | 307 | 343 | 365 | 561 | 247 |
| Multivariable-adjusted model | 1.41 (1.14 to 1.74) | 1.07 (0.86 to 1.32) | 1.00 | 1.34 (1.10 to 1.63) | 1.68 (1.35 to 2.08) |
| CVD mortality |  |  |  |  |  |
| No. of cases | 50 | 58 | 60 | 111 | 52 |
| Multivariable-adjusted model | 1.64 (0.94 to 2.84) | 1.20 (0.75 to 1.93) | 1.00 | 1.83 (1.20 to 2.78) | 1.87 (1.10 to 3.81) |

Multivariable-adjusted model: adjusted for age (continuous), sex (male or female), race/ethnicity (non-Hispanic white, non-Hispanic black, Hispanic, and other), educational level (less than high school, high school graduate or General Educational Development, and some college or above), family history of diabetes mellitus (yes or no), family history of heart disease (yes or no), history of diabetes (yes or no), history of heart disease (yes or no), history of cancer (yes or no), physical activity (0, 0.1-0.9, 1.0-3.4, 3.5-5.9, or ≥6 hours per week), drinking status (yes, no), smoking status (never smoker, former smoker, or current smoker), HEI-2015 scores (continuous), and body mass index (<21, 21-24.9, 25-29.9, 30-35, and ≥35).

Table S4 Sensitivity analysis of all-cause and cause-specific mortality according to sleep duration in NHANES by excluding the participants who died during the first year of follow-up.

|  | **Hazard ratio (95% CI)** | | | | |
| --- | --- | --- | --- | --- | --- |
|  | ≤5 | 6 | 7 | 8 | ≥9 |
| Person-years | 18397 | 29321 | 34133 | 32352 | 8447 |
| All-cause mortality |  |  |  |  |  |
| No. of cases | 162 | 187 | 225 | 314 | 123 |
| Multivariable-adjusted model | 1.42 (1.06 to 1.89) | 0.93 (0.69 to 1.26) | 1.00 | 1.31 (1.02 to 1.69) | 1.75 (1.27 to 2.42) |
| CVD mortality |  |  |  |  |  |
| No. of cases | 22 | 30 | 27 | 58 | 25 |
| Multivariable-adjusted model | 2.01 (0.83 to 4.84) | 1.38 (0.80 to 2.37) | 1.00 | 2.08 (1.14 to 3.79) | 2.06 (1.11 to 3.82) |

Multivariable-adjusted model: adjusted for age (continuous), sex (male or female), race/ethnicity (non-Hispanic white, non-Hispanic black, Hispanic, and other), educational level (less than high school, high school graduate or General Educational Development, and some college or above), family history of diabetes mellitus (yes or no), family history of heart disease (yes or no), history of diabetes (yes or no), history of heart disease (yes or no), history of cancer (yes or no), physical activity (0, 0.1-0.9, 1.0-3.4, 3.5-5.9, or ≥6 hours per week), drinking status (yes, no), smoking status (never smoker, former smoker, or current smoker), HEI-2015 scores (continuous), and body mass index (<21, 21-24.9, 25-29.9, 30-35, and ≥35).


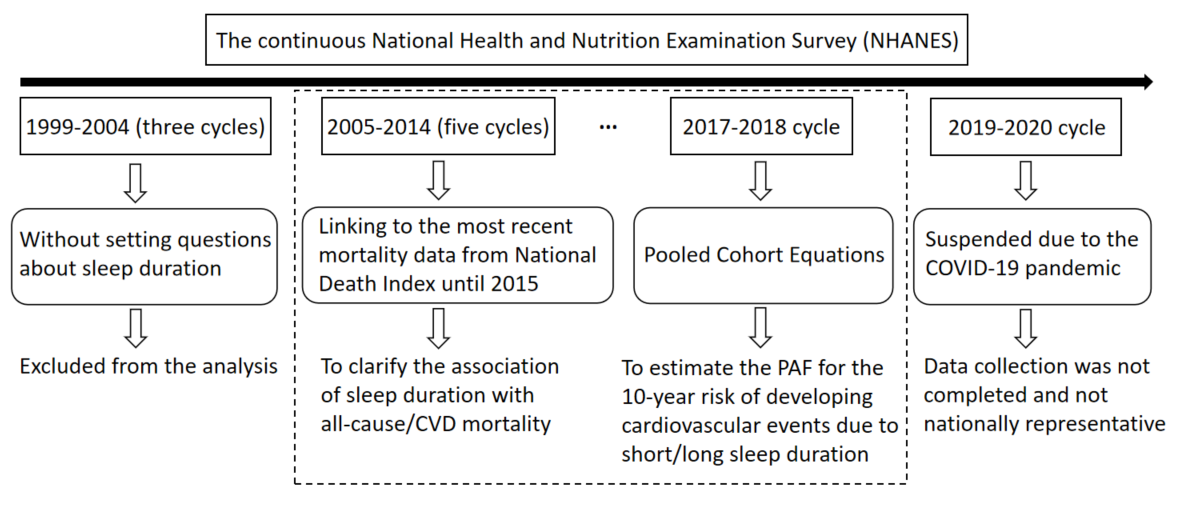


Figure S1 Flow chart of the study design.
